# Fellowship program directors and trainees across the United States find parental leave policies to be inconsistent, inaccessible, and inadequate

**DOI:** 10.1371/journal.pone.0260057

**Published:** 2021-11-17

**Authors:** Daniel Sabido Jamorabo, Amrin Khander, Vasilios Koulouris, Jeremy Eli Feith, William Matthew Briggs, Benjamin Dwight Renelus

**Affiliations:** 1 Department of Medicine, Stony Brook Medicine, Stony Brook, New York, United States of America; 2 Department of Obstetrics and Gynecology, Weill Cornell Medicine, New York, New York, United States of America; 3 Department of Medicine, Montefiore Medical Center, Bronx, New York, United States of America; 4 Department of Neurosciences, State University of New York-Binghamton, Binghamton, New York, United States of America; 5 Department of Biostatistics, New York-Presbyterian Brooklyn Methodist Hospital, Brooklyn, New York, United States of America; 6 Department of Medicine, Johns Hopkins Medicine, Baltimore, Maryland, United States of America; Indiana University, UNITED STATES

## Abstract

**Introduction:**

Determine the consistency, accessibility, and adequacy of parental leave policies for adult and pediatric medicine fellowship programs.

**Methods:**

We administered a 40-question survey to fellowship program directors (PDs) and trainees in adult and pediatric cardiology, hematology/oncology, gastroenterology, and pulmonology/critical care fellowship programs in the United States. We used Chi-square tests to compare proportions for categorical variables and t-tests to compare means for continuous variables.

**Results:**

A total of 190 PDs from 500 programs (38.0%) and 236 trainees from 142 programs (28.4%) responded. Most respondents did not believe that parental leave policies were accessible publicly (322/426; 75.6%), on password-protected intranet (343/426; 80.5%), or upon request (240/426; 56.3%). The PDs and trainees broadly felt that parental leave for fellows should be 5–10 weeks (156/426; 36.6%) or 11–15 weeks (165/426; 38.7%). A majority of PDs felt that there was no increased burden upon other fellows (122/190; 64.2%) or change in overall well-being (110/190; 57.9%). When asked about the biggest barrier to parental leave support, most PDs noted time constrains of fellowship (101/190; 53.1%) and the limited number of fellows (43/190; 22.6%). Trainees similarly selected the time constraints of training (88/236; 37.3%), but nearly one-fifth chose the culture in medicine (44/236; 18.6%). There were no statistically significant differences in answers based on the respondents’ sex, specialty, or subspecialty.

**Discussion:**

Parental leave policies are broadly in place, but did not feel these were readily accessible, standardized, or of optimum length. PDs and trainees noted several barriers that undermine support for better parental leave policies, including time constraints of fellowship, the limited number of fellows for coverage, and workplace culture. Standardization of parental leave policies is advisable to allow trainees to pursue fellowship training and care for their newborns without undermining their educational experiences.

## Introduction

The United States is one of only two countries worldwide that does not guarantee paid family leave [1]. The Family Medical Leave Act (FMLA) is a federally-appointed program that mandates 12 weeks of unpaid, job-protected parental leave for federal employees, but requires twelve months of employment prior to the request [2]. FMLA often defers to employers to regulate their employees’ family leave terms, especially in cases where trainees are new. In medical training, this often amounts to program or hospital leadership interpreting guidelines set forth from governing bodies to determine the available length of time that trainees can use towards delivery and care for their children [3]. The American Board of Internal Medicine (ABIM) and American Board of Pediatrics (ABP) have put forth guidelines for parental leave policies for trainees, however the Accreditation Council for Graduate Medical Education (ACGME) has not standardized these measures [4–6]. Per the ABIM, five weeks per year may be permitted for time away from training, which would include vacation and parental leave [4]. The ABP permits one month of time away from training; however, program directors may submit a petition to the ABP for waiver of up to two months of elective training for family leave [5]. The American Board of Medical Specialists (ABMS) recently adopted a parental leave policy which would allow for a minimum of six weeks of leave once during training, but this rule only applies to programs that are three years or longer [7]. Still, a review of formal leave policies across multiple graduate medical education (GME) programs across the country showed variability in their approaches [6] and dissatisfaction among trainees [8]. A recent review on parenthood during training identified trainees’ well-being as an important consideration for parental leave policies, but noted that many training programs were unprepared for situations wherein a trainee would need time off [9].

Various studies have examined parental leave policies in procedural residencies, including anesthesiology [10], general surgery [11], orthopedic surgery [12], and radiation oncology [13], among others, but there have been no studies focused on pediatric or adult medicine, particularly the subspecialties that involve the longest amount of training: hematology/oncology, cardiology, gastroenterology, and pulmonology/critical care. Our goal was to investigate the state of parental leave policies for such programs and to determine their accessibility to trainees, how consistent they were across programs, and how trainees’ education was impacted. We also wanted to assess the level of awareness that fellows and program directors in adult and pediatric subspecialties have about parental leave options during training as well as their views on how parental leave may affect clinical education and performance.

## Materials and methods

We carried out a cross-sectional survey of program directors (PDs) and trainees from adult and pediatric medicine subspecialty programs in cardiology, hematology/oncology, gastroenterology (GI), and pulmonology/critical care. We selected these specific subspecialties because they are the longest ones in both pediatric and adult medicine—three years each—and therefore would be more likely to include older trainees who have become new parents. Furthermore, targeting these fellowships would allow us to collect data from trainees who are in training for an extended period of time.

A search of existing literature search did not yield a validated questionnaire specific to our subspecialties of interest that we could adopt. We therefore reviewed parental leave-themed surveys carried out in other medical specialties [13–18] and adapted themes from other studies, in particular perceived barriers to clinical education, work-life balance, parenthood in medical training, and utility of parental leave. We then designed questions based upon these themes and piloted them with experienced program directors to ensure their applicability to adult medicine and pediatric subspecialties. We designed two 40-question surveys—one aimed at PDs and one at trainees—using feedback gained during the piloting stage (S1 and S2 Files).

The surveys were anonymous and voluntary. They included demographic questions including specialty and subspecialty choices, postgraduate year, location of training program, age, and sex of the participant. These demographic questions were followed by inquiries into the respondents’ knowledge about specific parental leave policies at their institutions, their length, and requirements when returning from leave. Both surveys asked respondents to answer questions about their perceived effect of parental leave upon clinical training and education. The trainees’ survey also included Likert Scale questions where trainees would subjectively rate their support at their institution. Space for free responses was included for trainees to voice any thoughts that were not addressed in the survey directly.

We obtained a list of PDs and coordinators from the American College of Graduate Medical Education (ACGME) website. We distributed the surveys to 500 programs (125 from each of the four subspecialties) using the Research Electronic Data Capture (REDCap) platform hosted at Weill Cornell Medical College. REDCap is a resource supported by the Weill Cornell Medicine Clinical and Translational Science Center and subsidized by National Institute of Health grant UL1-TR002384. This is an online server for collecting and organizing survey data and contact information. It allowed us to send secure emails with survey links unique to each email address and to collect all data anonymously and securely.

We emailed the PDs with reminder emails once every 7 days over the study period, which ran from 09/10/19 to 11/20/19. The trainee surveys were distributed from 01/27/20 to 03/16/2020 with reminder emails every 7 days, but due to the emerging COVID-19 pandemic we terminated our recruiting efforts. The surveys were voluntary and no incentives—financial or otherwise—were offered.

We used Chi-square tests to compare proportions for categorical variables, specifically responses from PDs in adult versus pediatric subspecialties. We ran t-tests to determine significance for numerical variables. All analyses were carried out in R version 3.6.5 (R Foundation for Statistical Computing, Vienna, Austria). The project was exempted by the New York-Presbyterian Institutional Review Board (IRB# 1465661–1).

## Results

A total of 190 PDs and 236 trainees filled out the survey. The response rate for the PDs was 38.0% (190/500 programs), while the trainees represented 142 different programs (142/500; 28.4%). Women comprised 87/190 (45.6%) of the PDs and 130/236 (55.1%) of the trainees. Approximately two-thirds of all respondents (273/426; 64.1%) were from adult medicine. Other baseline characteristics of the cohort are shown in Table 1.

**Table 1:** Baseline characteristics.

Variables		Program Director Responses (n = 190)	Percent (%)	Trainee Responses (n = 236)	Percent (%)
**Sex**	**Female**	87	45.6	130	55.1
	**Male**	103	54.4	106	44.9
**Specialty**	**Adult Medicine**	119	62.6	154	65.3
	**Pediatrics**	71	37.4	82	34.7
**Subspecialty**	**Cardiology**	47	24.7	62	26.3
	**Gastroenterology**	39	20.5	46	19.4
	**Hematology/Oncology**	57	30.0	70	29.7
	**Pulmonology and Critical Care**	47	24.7	58	24.6
**Region**	**Midwest**	52	27.4	53	22.5
	**Northeast**	70	36.8	99	41.9
	**South**	48	25.3	48	20.3
	**West**	20	10.5	36	15.3

**Fig 1:**
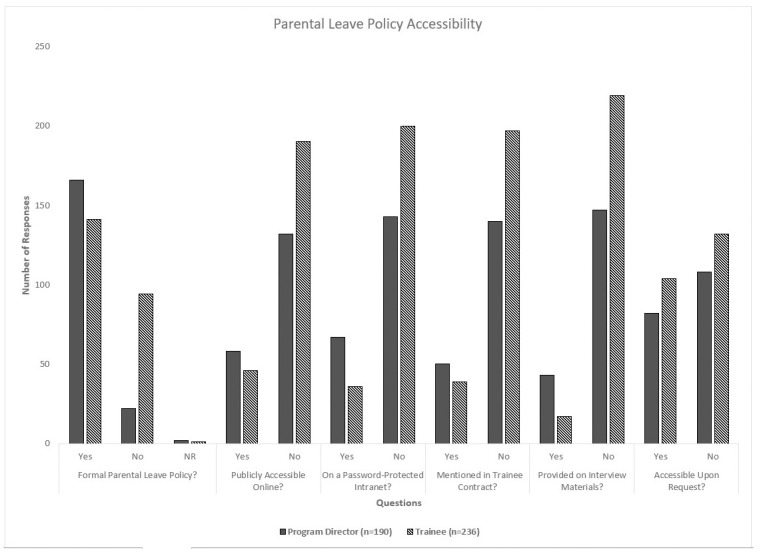
Responses on accessibility of parental leave policies.

**Fig 2:**
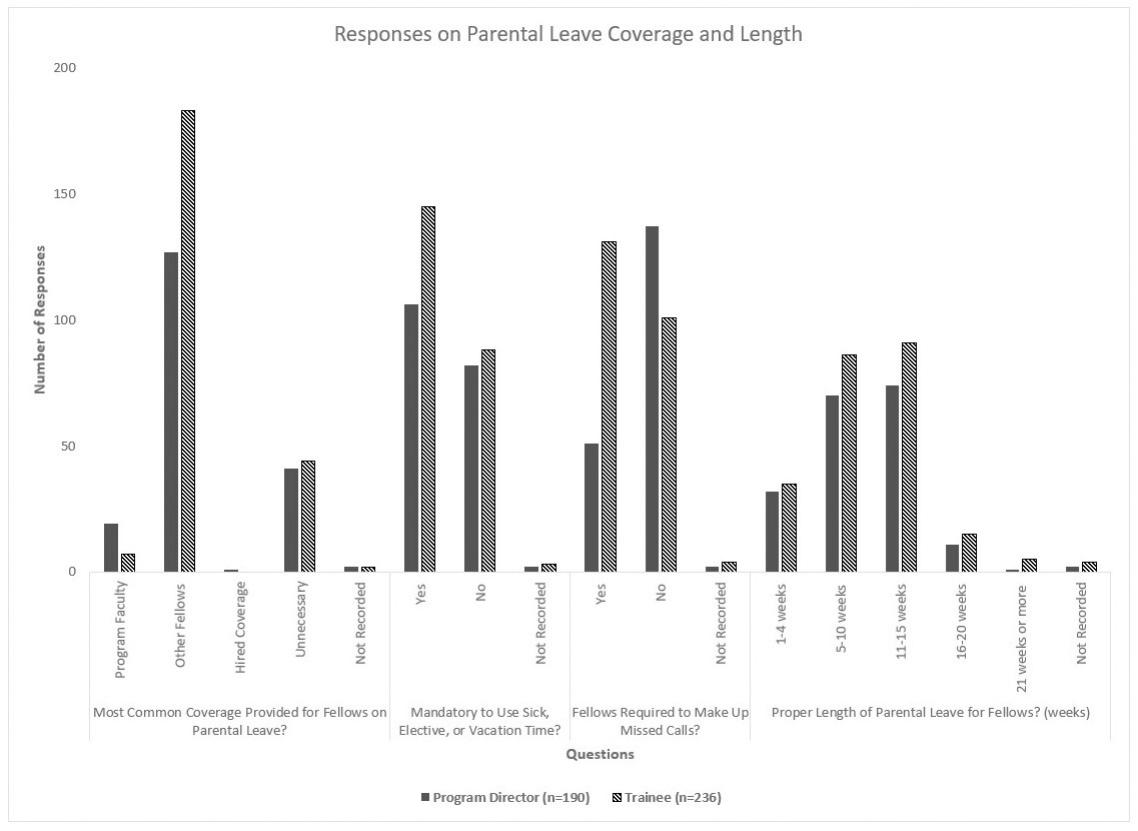
Responses on parental leave coverage and length.

Though a majority of respondents (307/426; 72.1%) answered that their program had a formal parental leave policy, most did not believe that such policies were accessible on a public webpage (322/426; 75.6%), on password-protected intranet (343/426; 80.5%), on trainees’ contracts (337/426; 79.1%), on fellowship interview materials (366/426; 85.9%), or upon request (240/426; 56.3%). Moreover, fellows on parental leave most commonly had their clinical rotations covered by their co-fellows (310/426; 72.8%) and were mandated to use sick, elective, or vacation time as part of their leave (251/426; 58.9%). While most of the PDs (137/190; 72.1%) replied that fellows were not required to make up calls missed during parental leave, over half of the trainees (131/236; 55.5%) answered the opposite. There were no statistically significant differences in answers based on the respondents’ sex, specialty, or subspecialty. The data for parental leave policy accessibility and coverage are summarized in Figs 1 and 2.

The PDs and trainees broadly felt that parental leave for fellows should be 5–10 weeks (156/426; 36.6%) or 11–15 weeks (165/426; 38.7%). PDs answered that fellows were most often offered 6 weeks of parental leave (77/190; 40.5%), with a large group saying 10 or more weeks were offered (51/190; 26.8%), for childbearing parents. In contrast, non-childbearing fellows were more often provided 2 weeks’ leave (54/190; 28.4%) and sometimes no leave (21/190; 11.1%). The trainees noted that the average length of parental leave offered for childbearing fellows was 5.3 weeks (median 6, SD 2.9) and for non-childbearing fellows was 2.4 weeks (median 2, SD 2.5), a difference that was statistically significant (p<0.001). There were no statistically significant differences in answers based on the respondents’ sex, specialty, or subspecialty.

**Fig 3:**
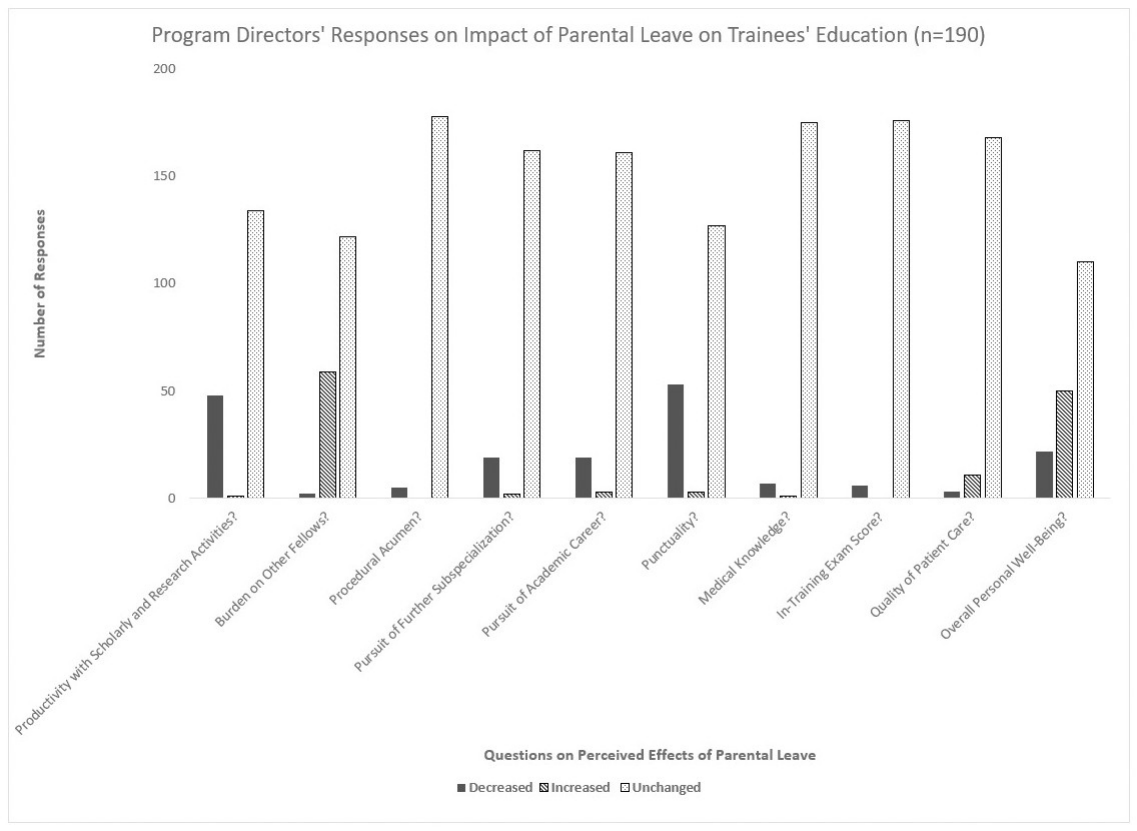
Program directors’ responses on parental leave impact on trainees’ education.

Most of the PD respondents did not believe that parental leave impacted their trainees’ research activities (134/190; 70.5%), procedural acumen (178/190; 93.7%), pursuit of further sub-specialization (162/190; 85.3%), or other aspects of their clinical performance. A majority likewise felt that there was no increased burden upon other fellows (122/190; 64.2%) or change in overall well-being (110/190; 57.9%). Trainees overall agreed with statements that said their program faculty and leadership were supportive of parental leave (156/426; 36.6%), approachable (150/426; 35.2%), and sensitive to childcare needs (151/426; 35.4%), but a large group also worried about burdening their co-fellows (178/426; 41.8%) and disagreed that the parental leave policies at their programs were adequate (105/426; 24.6%). In addition, most trainees answered that arranging childcare has been difficult during training (111/236; 47.0%) and that childcare needs limit research productivity (144/236; 61.0%). There were no statistically significant differences in answers based on the respondents’ sex, specialty, or subspecialty. The PD and trainee responses are provided in Fig 3 and Table 2.

**Table 2:** Likert scale survey questions (n = 231).

Survey Questions	Strongly Agree	Agree	Neutral/Not Applicable	Disagree	Strongly Disagree
Program Leadership and Faculty are Supportive of Parental Leave	68	88	39	29	7
Program Leadership and Faculty are Sensitive to Fellows’ Childcare Needs	55	96	51	23	6
I Feel Comfortable Approaching Program Leadership about Parental Leave and Childcare Needs	59	91	45	26	10
I Feel Pressure to Plan my Pregnancy or Parental Leave During Research and Elective Time	59	62	63	29	18
I Worry that Taking Parental Leave will Burden my Co-Fellows	83	95	26	21	6
My Co-Fellows are Supportive of Parental Leave	79	93	41	15	3
My Co-Fellows are Sensitive to my Childcare Needs	63	93	57	13	5
My Union has been Helpful in Advocating for Parental Leave	16	13	182	11	9
I Worry that my Work Schedule may Compromise my Pregnancy	36	70	100	20	5
I Feel that the Parental Leave Policy at my Program is Adequate	22	55	49	66	39
Lactation Rooms are Available 24/7 for Trainees	53	81	61	19	17
Lactation Rooms are Easily Accessible and Clean	37	60	86	26	22
Fellowship is the Best Stage of Training to have a Baby	21	49	84	53	24
Childcare Needs Discourage me from Pursuing Further Sub-Specialization	23	42	75	78	13
Childcare Needs make an Academic Career more Appealing	14	55	84	63	15
Childcare Needs make a Private Practice Career More Appealing	19	60	93	51	8
Childcare Needs Limit Research Productivity	49	95	69	18	0
Arranging Childcare has been Difficult While in Training	45	66	104	14	2
I Opted Primarily to Breastfeed while in Training	35	30	155	8	3
I Opted Primarily to Use Formula Feeds while in Training	7	15	149	34	26
I Suffered from Depression in the Post-Partum Period	4	18	147	40	22
I Had Adequate Access to Counseling in Post-Partum Period	5	26	180	14	6
Colleagues in my Field are Generally Supportive of New Parents and Parental Leave	43	107	56	20	5
I am Satisfied with the Mentorship I received about Balancing Training and Parenthood	10	55	100	52	14

**Fig 4:**
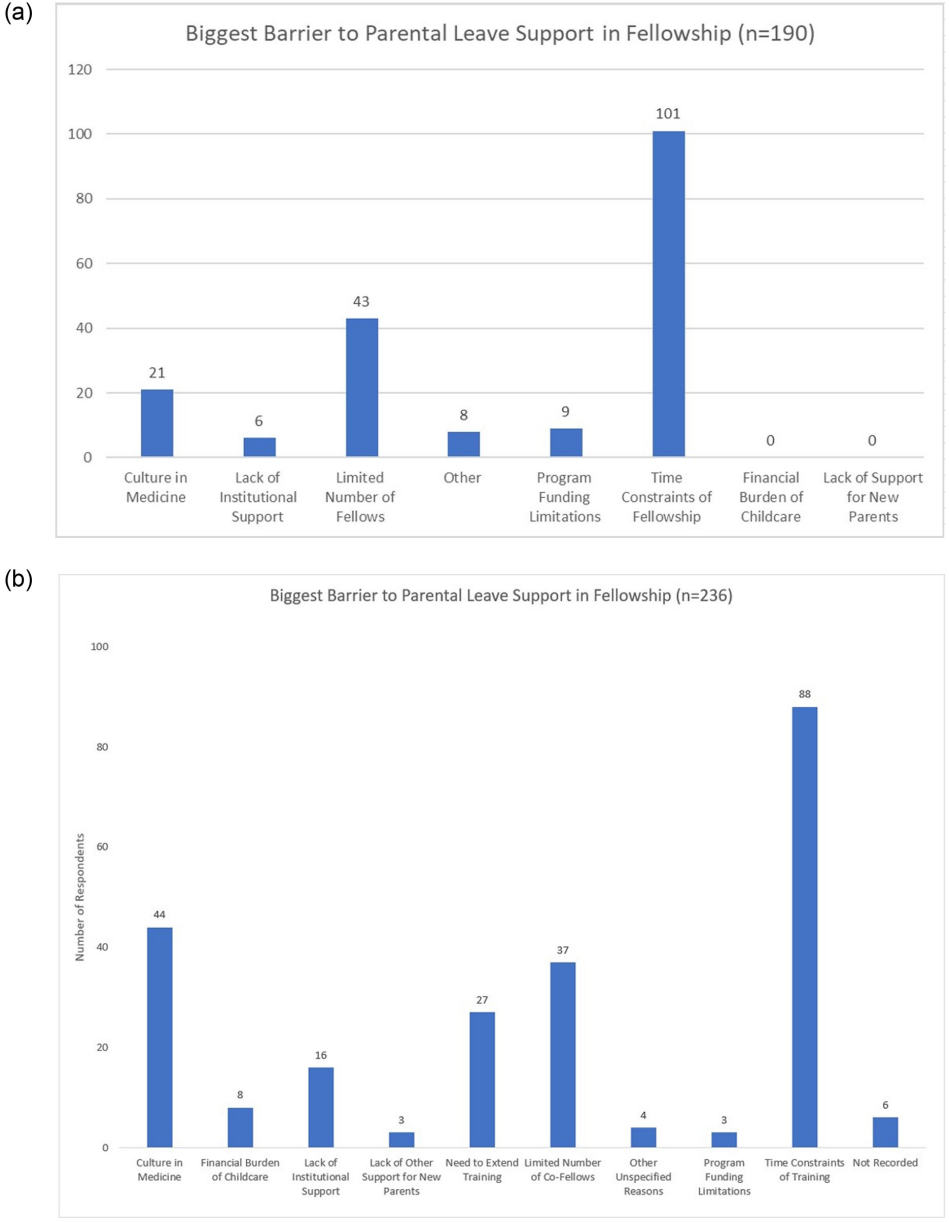
Biggest barrier to parental leave support in fellowship according to a) Program Directors and b) Trainees.

When asked about the single biggest barrier to parental leave support in fellowship, most PDs noted time constrains of fellowship (101/190; 53.1%) and the limited number of fellows (43/190; 22.6%). Trainees similarly selected the time constraints of training as the biggest barrier to parental leave support (88/236; 37.3%), but nearly one-fifth chose the culture in medicine (44/236; 18.6%) as the main challenge. The PD and trainee answers are illustrated in Fig 4a and 4b.

## Discussion

To our knowledge, this is the largest study on parental leave done on PDs and trainees in internal medicine fellowship programs. Our study findings describe the perceptions of program directors and trainees toward parental leave policies at a substantial number of adult and pediatric medicine fellowship programs. Though our respondents broadly answered that their programs had a parental leave policy in place, both PDs and trainees across all subspecialties did not believe that information on such policies was easily accessible or even included on fellows’ contracts. This finding is consistent with previous research showing that residents in various specialties are often unaware of parental leave policies at their programs [19]. Additionally, our respondents noted that parental leave often included sick, vacation, and research time, which suggests that at some programs the parental leave available is not so much a block of time independent of training requirements, but rather a realignment of a fellow’s schedule to have non-clinical rotations arranged in succession.

Our respondents came from the more procedural and lengthier subspecialties within both adult and pediatric medicine, so the PDs and trainees’ views on parental leave are not necessarily representative of other subspecialties or general internists and pediatricians. The PDs in our study listed time constraints and limited number of fellows as obstacles to supporting parental leave, which suggests that logistical issues concerning length of training and fellow coverage do affect the parental leave choices that fellows make and are offered. A recent retrospective cohort study of 31,878 Canadian women found that women physicians were more likely to delay their first childbirth several years after non-physician women, with specialists from both surgical and non-surgical fields delaying after family medicine practitioners [20]. Another survey of female residents across 25 specialties in 6 United States academic centers found that 274/447 (61%) of respondents chose to delay pregnancy due to lack of child care access, work schedules, and fear of burdening colleagues, among other reasons [21]. Both studies offer insight into challenges our respondents face when considering whether or not to have children, much less take parental leave, during fellowship training. Delaying pregnancy in subspecialties with longer training periods can also pose health concerns for trainees with regard to fertility and risk for high-risk pregnancies with advanced maternal age [16, 22, 23].

Parental leave guidelines are not uniform across medical residencies and fellowships, which can impact career choices if trainees fear they may have to delay graduation and board certification [24, 25]. We observed a difference in parental leave duration offered to childbearing and non-childbearing parents. Jolly, et al. found that male physicians were almost twice as likely as their female counterparts (85.6% versus 44.9%) to have domestic partners who were not employed full-time [26], which could partially explain the discrepancy we found if non-childbearing trainees opted to continue working. Our respondents’ overall preference for parental leave lasting 5–10 weeks or 11–15 weeks likewise differs from another survey that found that 619/844 (73%) physician mothers would have preferred up to 6 months of leave as opposed to the 5–12 weeks more commonly available [27]. Unlike the women surveyed who came from varied private and academic environments, including 138 residents (16%), our cohort is composed exclusively of people who work in training programs and for whom six months’ leave may be difficult to fit into a fellowship curriculum.

Others have written of the difficulty in bringing up family planning choices during residency and fellowship interviews [28]. Our findings were consistent with this as PDs and trainees alike reported that information on parental leave was generally not included in fellowship interview materials or employment contracts. The lack of clarity on parental leave policies before and during training may exacerbate cultural and social barriers to childbearing during training. Others have noted that lack of funding for maternity leave, cultural issues, and asynchrony between completing residency and starting fellowship are major challenges to a uniform parental leave policy [6]. SJ Finch has written about “anger and resentment” toward pregnant residents [29] and Adesoye, et al. reported feelings of workplace discrimination and burnout among new physician mothers who participated in an online support group [30]. PDs and trainees from our cohort largely cited the time constraints of fellowship as the major barrier to parental leave, though sizable groups also listed the limited number of fellows, the culture of medicine, and the potential need to extend training as other obstacles.

A major consideration is that programs may be left with inadequate guidance from state and local governments to help craft ACGME policies that can accommodate differences among specialties, such as procedural versus outpatient-based fields. Individual PDs or Designated Institutional Officers (DIOs) may not necessarily feel empowered or comfortable designing parental leave policies that they deem fit, particularly within a broader ACGME-accredited institution. For example, even when a governing board has laid out guidelines on parental leave, training programs may not necessarily implement changes, as noted in a recent survey of PDs in obstetrics and gynecology [31]. Lumpkin, et al. found that ACGME specialty leave policies for 24 residency and fellowship programs offer less than the FMLA-mandated 12 weeks to their trainees, possibly to avoid significant delays in board certification [25]. A recent study noted that 59/218 (27%) of new resident mothers cited a desire to not extend their training as the main consideration for choosing or refusing maternity leave [32]. In our own study, there is a prevailing sense among PDs and trainees that parental leave does not negatively impact fellows’ training and that parental leave for fellows should be longer. A majority of PDs felt that there was no change in the burden placed on other fellows and that new parents were generally not required to make up missed calls, yet they, along with most trainees (127/190 PDs and 183/236 trainees), replied that most call coverage for parental leave went to other fellows. This discordance may be the result of PDs and trainees coming from different programs and could potentially be resolved in future studies.

We call upon the ACGME, the American Board of Internal Medicine, and the American Board of Pediatrics to recommend standardized parental leave policies in light of continued discrepancies within and between specialties as reported in our findings. One could argue that the variability in parental leave policies across subspecialties may reflect adaptability to the needs of different training programs, but this would be inconsistent with the fact that ACGME has not articulated that parental leave policies should be left to the discretion of individual programs or specialties [3, 6, 18, 33–36]. The American Board of Obstetrics and Gynecology (ABOG) has recently extended permissible leaves of absence—including for parental leave—during residency and fellowship to twelve weeks in any given year of training [37], which is well within the range advised by the respondents in our study. This policy is effective immediately and required of all ACGME-accredited obstetrics and gynecology programs. It is an example of the broad positive impact that governing boards can have upon medical education with clear directives.

We also encourage other physician organizations to help craft such changes to benefit all programs and trainees. PDs have highlighted opportunities for improvement, including equitable leave offerings for childbearing and non-childbearing trainees, addressing barriers to parental leave, and improving access to leave policies for applicants and fellows. The lack of statistically significant differences in responses based on adult medicine and pediatrics specialists underscores how widespread concerns about parental leave are. Most PDs in our cohort did not feel parental leave adversely affects the ultimate educational experience, so addressing the logistics of coverage, board certification eligibility, and workplace culture are paramount. National specialty and subspecialty organizations could be powerful advocates in laying out guidelines on parental leave, and how to facilitate board certification and transition to fellowship while minimizing the extra time needed to meet training certification requirements.

The limitations of our study include the modest response rate of 38.0% for PDs and 236 trainees from 500 total programs. We excluded subspecialties including sports medicine, endocrinology, rheumatology, among others, which tend to be shorter, less procedural, and have more balanced gender ratios than those we targeted. It is possible that self-selection provided a sample of PDs and trainees less likely to find parental leave policies to be satisfactory than the general population of subspecialists. We did not fully capture sexual minorities in our survey and our phrasing in questions regarding non-childbearing and childbearing parents, particularly regarding “male” and “female” parents may not have prompted respondents—especially PDs—to consider transgender trainees and their parental leave concerns. The difference in leave patterns for childbearing and non-childbearing parents may not account for same-sex couples or even for heteronormative ones where the non-childbearing trainee’s partner is also working full-time. We did not ask specifically about adoptions, though we assumed that parental leave considerations for this would be similar to those of non-childbearing parents.

The PDs responses on how parental leave affects their trainees’ education and skills cannot tease out the effect of taking leave versus the effect of having children. This is due to their answering questions intentionally meant to gauge their attitudes toward parental leave. Finally, our survey aimed to capture the perceptions of PDs and trainees regarding accessibility, utility, and challenges involved with parental leave policies. Neither group is necessarily knowledgeable about or empowered to design their institutions’ parental leave policies unlike, for instance a DIO. Still, they would be the obvious sources for information to which prospective applicants and fellows would turn for information, and their answers reflect a general sense that current parental leave policies are in need of improvement and transparency. Finally, our study focused on parental leave, but another potential area of study would be balancing childcare with clinical training once parental leave is finished. This was beyond the scope of our survey, but could be of interest since clinical duties may prevent physicians from pursuing personal hobbies and spending time with their children, which could in turn affect their morale and job satisfaction [38].

## Conclusion

The findings of the survey include a spectrum of subspecialties and provide a broad view of the state of parental leave for our trainees and opportunities for improvement. We hope that our findings help spur programs and physician organizations to devise and implement constructive changes to parental leave policies for trainees in the near future.

## Supporting information

S1 File Survey instrument administered to program directors. (PDF)

S2 File Survey instrument administered to trainees. (PDF)
