# Supplementary material for: Fellowship program directors and trainees across the United States find parental leave policies to be inconsistent, inaccessible, and inadequate
Source: PLoS One. 2021 Nov 17;16(11):e0260057. doi: 10.1371/journal.pone.0260057 (PMC8598025; doi:10.1371/journal.pone.0260057)
Supplement: S1 File — (PDF) [file pone.0260057.s001.pdf]

Parental Leave and Subspecialty Training

Thank you for participating in our survey! Our project aims to identify the variety of parental leave policies in subspecialty training programs across the U.S.

All responses are confidential and anonymous. Your participation is much appreciated.

Select your specialty

◯ Internal Medicine
◯ Pediatrics

Select your subspecialty

◯ Cardiology
◯ Gastroenterology
◯ Hematology/Oncology
◯ Pulmonology/Critical Care

Select your gender

◯ Male
◯ Female
◯ Other

Select your age range

◯ 30-39 years
◯ 40-49 years
◯ 50-59 years
◯ 60 years and above

Select the number of years you have been out of training

◯ Less than 5
◯ 5-10
◯ 11-15
◯ 16-20
◯ 21-25
◯ 26 or more

Select the number of years you have served as the program director at your current program

◯ Less than 5
◯ 5-10
◯ 11-15
◯ 16-20
◯ 21-25
◯ 26 or more

What is your marital status?

◯ Single
◯ Unmarried but cohabitating with partner
◯ Married
◯ Divorced
◯ Widowed

Is your significant other employed?

◯ Yes
◯ No
◯ Not Applicable

If your significant is employed, is this from home, self-employed, part-time, or full-time?

◯ From home
◯ Self-employed
◯ Part-time
◯ Full-time
◯ More than one of the above
◯ Not Applicable

How many children (if any) do you have?

◯ 0
◯ 1
◯ 2
◯ 3
◯ 4
◯ 5
◯ 6
◯ 7 or more

At what level of your training did you have your first child?

◯ Before medical school
◯ During medical school
◯ During residency
◯ During fellowship
◯ As an attending
◯ Not Applicable

At what level of your training did you have your last child?

◯ Before medical school
◯ During medical school
◯ During residency
◯ During fellowship
◯ As an attending
◯ Not Applicable

Did you take parental leave during fellowship?

◯ Yes
◯ No

If so, for how many weeks?

◯ 0
◯ 1
◯ 2
◯ 3
◯ 4
◯ 5
◯ 6
◯ 7 or more
◯ Not Applicable

Did you have to delay graduation due to parental leave?

◯ Yes
◯ No

Select the primary training site for your program

◯ University Hospital
◯ Community Affiliate
◯ Veteran Affairs Medical Center

In which region is your training site located?

◯ Northeast
◯ Midwest
◯ South
◯ West

How many male faculty attendings are in your program?

◯ 0
◯ 1
◯ 2
◯ 3
◯ 4
◯ 5
◯ 6
◯ 7
◯ 8
◯ 9
◯ 10 or more

How many female faculty attendings are in your program?

◯ 0
◯ 1
◯ 2
◯ 3
◯ 4
◯ 5
◯ 6
◯ 7
◯ 8
◯ 9
◯ 10 or more

How many male fellows are in your program?

◯ 0
◯ 1
◯ 2
◯ 3
◯ 4
◯ 5
◯ 6
◯ 7
◯ 8
◯ 9
◯ 10 or more

How many of your male fellows have children?

◯ 0
◯ 1
◯ 2
◯ 3
◯ 4
◯ 5
◯ 6
◯ 7
◯ 8
◯ 9
◯ 10 or more

How many female fellows are in your program?

◯ 0
◯ 1
◯ 2
◯ 3
◯ 4
◯ 5
◯ 6
◯ 7
◯ 8
◯ 9
◯ 10 or more

How many of your female fellows have children?

◯ 0
◯ 1
◯ 2
◯ 3
◯ 4
◯ 5
◯ 6
◯ 7
◯ 8
◯ 9
◯ 10 or more

Has your training program granted any of the following types of leave within the past 5 years?

☐ Parental/maternity leave
☐ Sick leave
☐ Psychiatric leave
☐ Military leave
☐ Other
☐ None of the above

Are you aware of the ACGME, American Board of Internal Medicine, or American Board of Pediatrics allowances for parental leave?

◯ Yes
◯ No

Does your training program have a formally outlined policy regarding parental leave?

◯ Yes
◯ No

If not, is such a policy being planned?

◯ Yes
◯ No
◯ Not Applicable

Where can these policies be accessed?

☐ Publicly accessible online
☐ Password-protected intranet
☐ Employment contract
☐ Fellowship interview materials
☐ On request
☐ Not Applicable

What is the MOST COMMON coverage provided for fellows who take parental leave?

◯ Other fellows
◯ Moonlighters, hired coverage
◯ Attending physician
◯ Coverage typically not necessary

How many weeks are fellows GIVEN for childbearing (i.e. maternity) leave?

◯ 0
◯ 1
◯ 2
◯ 3
◯ 4
◯ 5
◯ 6
◯ 7
◯ 8
◯ 9
◯ 10 or more

How many weeks are fellows GIVEN for non-childbearing or paternity leave?

◯ 0
◯ 1
◯ 2
◯ 3
◯ 4
◯ 5
◯ 6
◯ 7
◯ 8
◯ 9
◯ 10 or more

How many weeks do fellows typically TAKE for childbearing (i.e. maternity) leave?

◯ 0
◯ 1
◯ 2
◯ 3
◯ 4
◯ 5
◯ 6
◯ 7
◯ 8
◯ 9
◯ 10 or more

How many weeks do fellows typically TAKE for non-childbearing or paternity leave?

◯ 0
◯ 1
◯ 2
◯ 3
◯ 4
◯ 5
◯ 6
◯ 7
◯ 8
◯ 9
◯ 10 or more

What percentage of fellows who take parental leave extend their training?

◯ 0-20%
◯ 21-40%
◯ 41-60%
◯ 61-80%
◯ 81-100%

Are they required to use available sick, elective, or vacation time as part of parental leave?

◯ Yes
◯ No

Are they required to make up missed call shifts?

◯ Yes
◯ No

Does your program or department provide lactation rooms for nursing trainees?

◯ Yes
◯ No

If these rooms are not provided by the program or department, are they available in the hospital?

◯ Yes
◯ No
◯ I don't know

Are these rooms easily accessible (location, cleanliness, 24hr availability)?

◯ Yes
◯ No
◯ I don't know

In which of the following do you feel that having a baby affects a MALE (or non-childbearing) fellow's work quality?

	Increased	Unchanged	Decreased
Scholarly activities	◯	◯	◯
Burden on other fellows	◯	◯	◯
Proficiency at procedures	◯	◯	◯
Pursuit of further subspecialty training	◯	◯	◯
Pursuit of an academic career	◯	◯	◯
Punctuality	◯	◯	◯
Medical knowledge	◯	◯	◯
In-training exam scores	◯	◯	◯
Patient care	◯	◯	◯
Overall well-being	◯	◯	◯

In which of the following do you feel that having a baby affects a FEMALE (or childbearing) fellow's work quality?

	Increased	Unchanged	Decreased
Scholarly activities	◯	◯	◯
Burden on other fellows	◯	◯	◯
Proficiency at procedures	◯	◯	◯
Pursuit of further subspecialty training	◯	◯	◯
Pursuit of an academic career	◯	◯	◯
Punctuality	◯	◯	◯
Medical knowledge	◯	◯	◯
In-training exam scores	◯	◯	◯

Patient care	◯	◯	◯
Overall well-being	◯	◯	◯

How long do you feel parental leave for fellows should be?

◯ 1-4 weeks
◯ 5-10 weeks
◯ 11-15 weeks
◯ 16-20 weeks
◯ 21 weeks or more

Which of the following is the BIGGEST BARRIER to supporting parental leave for fellows?

◯ Time constraints of fellowship
◯ Program funding limitations
◯ Limited number of fellows
◯ Culture of parental leave in medicine
◯ Financial burden of childcare
◯ Lack of institutional support
◯ Lack of support for new parents
◯ Other

Which of the following is the SECOND BIGGEST BARRIER to supporting parental leave for fellows?

◯ Time constraints of fellowship
◯ Program funding limitations
◯ Limited number of fellows
◯ Culture of parental leave in medicine
◯ Financial burden of childcare
◯ Lack of institutional support
◯ Lack of support for new parents
◯ Other

Which of the following is the THIRD BIGGEST BARRIER to supporting parental leave for fellows?

◯ Time constraints of fellowship
◯ Program funding limitations
◯ Limited number of fellows
◯ Culture of parental leave in medicine
◯ Financial burden of childcare
◯ Lack of institutional support
◯ Lack of support for new parents
◯ Other
