# Supplementary material for: Fellowship program directors and trainees across the United States find parental leave policies to be inconsistent, inaccessible, and inadequate
Source: PLoS One. 2021 Nov 17;16(11):e0260057. doi: 10.1371/journal.pone.0260057 (PMC8598025; doi:10.1371/journal.pone.0260057)
Supplement: S2 File — (PDF) [file pone.0260057.s002.pdf]

Trainee Perspectives on Parental Leave

Thank you very much for participating in our 5-minute survey! Our project aims to elucidate how parental leave policies impact trainees' educational experience while in fellowship. We hope that your responses will help us advocate for parents in fellowship training across the country.

ALL responses are CONFIDENTIAL and ANONYMOUS. Your participation is greatly appreciated!

Please select your specialty:

◯ Internal Medicine
◯ Pediatrics

Please select your subspecialty:

◯ Cardiology
◯ Gastroenterology
◯ Hematology/Oncology
◯ Pulmonology/Critical Care

Please select your year of fellowship training:

◯ First-year
◯ Second-year
◯ Third-year
◯ Fourth-year

In which region is your training site located?

◯ Northeast
◯ Midwest
◯ South
◯ West

Please select the primary training site for your program:

◯ University Hospital
◯ Community Affiliate
◯ Veteran Affairs Medical Center

Are you part of a union for trainees?

◯ Yes
◯ No

What is your age?

____

Please select your sex:

◯ Female
◯ Male
◯ Other

Please select your race/ethnicity:

☐ American Indian/Native American
☐ Asian
☐ Black/African
☐ Hispanic/Latin American
☐ White/Caucasian
☐ Group(s) not listed above
☐ I identify with multiple options listed above

Please select your marital status:

◯ Single
◯ Unmarried but cohabitating with partner
◯ Married
◯ Divorced
◯ Widowed

Is your significant other employed?

◯ Yes
◯ No
◯ Not Applicable

If your significant other is employed, is this from home, self-employed, part-time, or full-time?

◯ From home
◯ Self-employed
◯ Part-time
◯ Full-time
◯ More than one of the above
◯ Not Applicable

How many fellows total are in your program?

____

How many children (if any) do you have?

◯ 0
◯ 1
◯ 2
◯ 3
◯ 4
◯ 5
◯ 6
◯ 7 or more

At what level of your training did you have your first child?

◯ Before medical school
◯ During medical school
◯ During residency
◯ During fellowship
◯ Not Applicable

At what level of your training did you have your last child?

◯ Before medical school
◯ During medical school
◯ During residency
◯ During fellowship
◯ Not Applicable

Are you aware of the ACGME, American Board of Internal Medicine, or American Board of Pediatrics allowances for parental leave?

◯ Yes
◯ No

Does your training program have a formally outlined policy regarding parental leave?

◯ Yes
◯ No

If not, is such a policy being planned?

◯ Yes
◯ No
◯ Not Applicable

Where can these policies be accessed?

☐ Publicly accessible online
☐ Password-protected intranet
☐ Employment contract
☐ Fellowship interview materials
☐ On request
☐ Not Applicable

Did you take parental leave during fellowship?

◯ Yes
◯ No
◯ Not Applicable

If so, for how many weeks?

◯ 0
◯ 1
◯ 2
◯ 3
◯ 4
◯ 5
◯ 6
◯ 7 or more
◯ Not Applicable

How many weeks are fellows OFFERED for childbearing (i.e. maternity) leave?

◯ 0
◯ 1
◯ 2
◯ 3
◯ 4
◯ 5
◯ 6
◯ 7
◯ 8
◯ 9
◯ 10 or more

How many weeks are fellows OFFERED for non-childbearing or paternity leave?

◯ 0
◯ 1
◯ 2
◯ 3
◯ 4
◯ 5
◯ 6
◯ 7
◯ 8
◯ 9
◯ 10 or more

How many weeks do fellows typically TAKE for childbearing (i.e. maternity) leave?

◯ 0
◯ 1
◯ 2
◯ 3
◯ 4
◯ 5
◯ 6
◯ 7
◯ 8
◯ 9
◯ 10 or more

How many weeks do fellows typically TAKE for non-childbearing or paternity leave?

◯ 0
◯ 1
◯ 2
◯ 3
◯ 4
◯ 5
◯ 6
◯ 7
◯ 8
◯ 9
◯ 10 or more

Did/will you have to delay graduation due to parental leave?

◯ Yes
◯ No
◯ Not Applicable

From which of the following was parental leave taken? (check all that apply)

☐ Clinical time
☐ Research/elective time
☐ Family and medical leave (FMLA)
☐ Not Applicable

Are fellows required to use available sick, elective, or vacation time as part of parental leave?

◯ Yes
◯ No

Which is the MOST COMMON coverage provided for fellows who take parental leave?

◯ Other fellows
◯ Moonlighters, hired coverage
◯ Attending physician
◯ Coverage typically not necessary

Are they required to make up missed call shifts?

◯ Yes
◯ No

Do you wish you had had children at a different stage of your career?

◯ Yes, earlier
◯ Yes, later
◯ No
◯ Not applicable

How long do you feel parental leave for fellows should be?

◯ 1-4 weeks
◯ 5-10 weeks
◯ 11-15 weeks
◯ 16-20 weeks
◯ 21 weeks or more

Please select your answers below:

	Strongly Agree	Agree	Neutral/Not Applicable	Disagree	Strongly Disagree
Program faculty and leadership are supportive of parental leave	○	○	○	○	○
Program faculty and leadership are sensitive to fellows' childcare needs	○	○	○	○	○
I feel comfortable approaching my program's leadership about parental leave and childcare needs	○	○	○	○	○
I feel pressure to plan my pregnancy or parental leave during research and elective time	○	○	○	○	○
I worry that taking parental leave will burden my co-fellows	○	○	○	○	○
My co-fellows are supportive of parental leave	○	○	○	○	○
My co-fellows are sensitive to my childcare needs	○	○	○	○	○
My union has been helpful in advocating for parental leave	○	○	○	○	○
I worry that my work schedule may compromise my pregnancy	○	○	○	○	○
I feel that the parental leave policy at my program is adequate	○	○	○	○	○
Lactation rooms are available 24/7 for trainees	○	○	○	○	○
Lactation rooms are easily accessible and clean	○	○	○	○	○
Fellowship is the best stage of training to have a baby	○	○	○	○	○

Please select your answers below:

	Strongly Agree	Agree	Neutral/Not Applicable	Disagree	Strongly Disagree
Childcare needs discourage me from pursuing further sub-specialization	◯	◯	◯	◯	◯
Childcare needs make a career in academia more appealing	◯	◯	◯	◯	◯
Childcare needs make a private practice career more appealing	◯	◯	◯	◯	◯
Childcare needs limit research productivity	◯	◯	◯	◯	◯
Arranging childcare has been difficult while in training	◯	◯	◯	◯	◯
I opted primarily to breastfeed while in training	◯	◯	◯	◯	◯
I opted primarily to use formula feeds while in training	◯	◯	◯	◯	◯
I suffered from depression in the post-partum period	◯	◯	◯	◯	◯
I had adequate access to counseling in the post-partum period	◯	◯	◯	◯	◯
Colleagues in my field are generally supportive of new parents and parental leave	◯	◯	◯	◯	◯
I am satisfied with the mentorship I received about balancing training and parenthood	◯	◯	◯	◯	◯

Which of the following is the BIGGEST BARRIER to supporting parental leave for fellows?

◯ Time constraints of fellowship
◯ Program funding limitations
◯ Limited number of fellows
◯ Culture of parental leave in medicine
◯ Financial burden of childcare
◯ Lack of institutional support
◯ Lack of support for new parents
◯ Likelihood of extending training period
◯ Other

Which of the following is the SECOND BIGGEST BARRIER to supporting parental leave for fellows?

◯ Time constraints of fellowship
◯ Program funding limitations
◯ Limited number of fellows
◯ Culture of parental leave in medicine
◯ Financial burden of childcare
◯ Lack of institutional support
◯ Lack of support for new parents
◯ Likelihood of extending training period
◯ Other

Which of the following is the THIRD BIGGEST BARRIER to supporting parental leave for fellows?

◯ Time constraints of fellowship
◯ Program funding limitations
◯ Limited number of fellows
◯ Culture of parental leave in medicine
◯ Financial burden of childcare
◯ Lack of institutional support
◯ Lack of support for new parents
◯ Likelihood of extending training period
◯ Other

Please share any other thoughts on parental leave in training that may not have been addressed above: ______________________________
